# Changes in free polyamine levels, expression of polyamine biosynthesis genes, and performance of rice cultivars under salt stress: a comparison with responses to drought

**DOI:** 10.3389/fpls.2014.00182

**Published:** 2014-05-08

**Authors:** Phuc T. Do, Oliver Drechsel, Arnd G. Heyer, Dirk K. Hincha, Ellen Zuther

**Affiliations:** ^1^Infrastructure Group Transcript Profiling, Max-Planck-Institute of Molecular Plant PhysiologyPotsdam, Germany; ^2^Department of Plant Biotechnology, Institute of Biology, University of StuttgartStuttgart, Germany

**Keywords:** polyamines, salt stress, drought stress, gene expression, rice, natural variety

## Abstract

Soil salinity affects a large proportion of rural area and limits agricultural productivity. To investigate differential adaptation to soil salinity, we studied salt tolerance of 18 varieties of *Oryza sativa* using a hydroponic culture system. Based on visual inspection and photosynthetic parameters, cultivars were classified according to their tolerance level. Additionally, biomass parameters were correlated with salt tolerance. Polyamines have frequently been demonstrated to be involved in plant stress responses and therefore soluble leaf polyamines were measured. Under salinity, putrescine (Put) content was unchanged or increased in tolerant, while dropped in sensitive cultivars. Spermidine (Spd) content was unchanged at lower NaCl concentrations in all, while reduced at 100 mM NaCl in sensitive cultivars. Spermine (Spm) content was increased in all cultivars. A comparison with data from 21 cultivars under long-term, moderate drought stress revealed an increase of Spm under both stress conditions. While Spm became the most prominent polyamine under drought, levels of all three polyamines were relatively similar under salt stress. Put levels were reduced under both, drought and salt stress, while changes in Spd were different under drought (decrease) or salt (unchanged) conditions. Regulation of polyamine metabolism at the transcript level during exposure to salinity was studied for genes encoding enzymes involved in the biosynthesis of polyamines and compared to expression under drought stress. Based on expression profiles, investigated genes were divided into generally stress-induced genes (*ADC2, SPD/SPM2, SPD/SPM3*), one generally stress-repressed gene (*ADC1*), constitutively expressed genes (*CPA1, CPA2, CPA4, SAMDC1, SPD/SPM1*), specifically drought-induced genes (*SAMDC2, AIH*), one specifically drought-repressed gene (*CPA3*) and one specifically salt-stress repressed gene (*SAMDC4*), revealing both overlapping and specific stress responses under these conditions.

## Introduction

Cultivation of rice (*Oryza sativa* L.) is limited by environmental stresses, of which salinity and drought represent some of the most devastating ones. Erosion, soil degradation and salinization affect approximately 3.6 billion of the world's 5.2 billion ha of dryland used for agriculture (Riadh et al., 2010). 10% of the land surface (950 Mha) and 50% of all irrigated land (230 Mha) are salt-affected (Ruan et al., 2010). Global annual losses from soil salinity are estimated at US$12 billion (Qadir et al., 2008). Rice (*Oryza sativa* L.) is considered to be moderately sensitive to salinity (Akita and Cabuslay, 1990) with a clear distinction between initial effects of salinity and long-term effects that result from the accumulation of salt in expanded leaves (Yeo et al., 1991). Salt stress causes reduction in leaf expansion, relative growth rate (Akita and Cabuslay, 1990) and photosynthesis (Nakamura et al., 2002; Cha-Um et al., 2006), as well as enhanced senescence (Lutts et al., 1996). Three major processes have been considered to participate in protection against high cytosolic Na^+^: (1) the minimization of Na^+^ entry into cells; (2) the compartmentation of Na^+^ into the vacuole; and (3) the increased efflux of Na^+^ out of the cell driven by specific ion transporters (Chinnusamy et al., 2005).

Polyamines are involved in a wide range of biological processes, including growth, development and programmed cell death (Galston and Sawhney, 1990; Bouchereau et al., 1999; Kaur-Sawhney et al., 2003; Moschou and Roubelakis-Angelakis, 2013), as well as abiotic stress responses (for recent reviews see Alcázar et al., 2010; Gill and Tuteja, 2010; Hussain et al., 2011; Gupta et al., 2013). Nevertheless, reported responses of polyamines to salt stress are often contradictory, even within one species. In rice, either a decrease of putrescine (Put) and/or spermidine (Spd) (Lin and Kao, 1995; Maiale et al., 2004), or of all three major polyamines (Prakash et al., 1988), but also a salt-induced increase of Put (Basu et al., 1988; Basu and Ghosh, 1991), Spd, and spermine (Spm) (Katiyar and Dubey, 1990; Maiale et al., 2004) has been reported. Also a differential modification of polyamines upon salt stress depending on the tolerance level was described (Krishnamurthy and Bhagwat, 1989). Considering water stress in rice in general, including osmotic stress, dehydration, and withholding water, accumulation of polyamines was reported for most scenarios (Capell et al., 2004) but also a decrease of Put with a parallel increase of Spm (Do et al., 2013). Modifications of polyamines in response to osmotic stress were considered to be affected by dose and time of treatment (Lefèvre et al., 2001). A transcriptome analysis of polyamine over-accumulators revealed that endogenous polyamines participate in stress signaling through a crosstalk with abscisic acid (ABA), Ca^2+^ signaling and other hormonal pathways in plant defense and development (Marco et al., 2011).

Positively charged polyamines are able to interact with negatively charged molecules, such as nucleic acids, acidic phospholipids, proteins, and cell wall components (Martin-Tanguy, 2001; Kakkar and Sawhney, 2002). The physiological function of the various polyamines in stress response is not resolved yet, but their involvement in protein phosphorylation, conformational transitions of DNA (Martin-Tanguy, 2001), maintenance of ion balance, radical scavenging and prevention of senescence, stabilization of membranes (Bouchereau et al., 1999), and gene regulation by enhancing DNA-binding activity of transcription factors (Panagiotidis et al., 1995) was shown and a role as compatible solutes is discussed. Additionally, polyamines affect ion channel conductivity due to their positive charge and are able to block vacuolar channels, e.g., calcium channels (Hussain et al., 2011).

Polyamine catabolism in the apoplast is a common mechanism within reactive oxygen species (ROS) signaling (Pottosin et al., 2014). Furthermore Spd and Spm were described as nitric oxide (NO) inducers in plants which are part of a complex network also containing ABA and H_2_O_2_ (Hussain et al., 2011; Pottosin et al., 2014). In addition, polyamines are major players in the turnover of nitrogenous compounds in plants under optimal as well as stress conditions (Moschou et al., 2012).

Put biosynthesis either from ornithine or indirectly from arginine via agmatine is catalyzed by ornithine (ODC; EC 4.1.1.17) and arginine decarboxylase (ADC; EC 4.1.1.19), respectively. Agmatine is then sequentially converted to Put by agmatine iminohydrolase (AIH; EC3.5.3.12) and N-carbamoylputrescine amidohydrolase (CPA; EC 3.5.1.53). Spd and Spm are synthesized from Put by the addition of aminopropyl groups, transferred from decarboxylated S-adenosylmethionine (SAM), which is produced from SAM by S-adenosylmethionine decarboxylase (SAMDC; EC 4.1.1.50). Spd synthase (SPD; EC 2.5.1.16) and Spm synthase (SPM; EC 2.5.1.22) catalyze the final steps of the Spd and Spm synthesis. Polyamine metabolism and transport have been recently reviewed in Gupta et al. (2013), and the integration with other metabolic networks was shown in *Arabidopsis* (Bitrián et al., 2012).

An induction of the transcript level and/or activity of ADC could be shown for rice (Chattopadhyay et al., 1997) as well as for other species (Mo and Pua, 2002; Urano et al., 2004; Hao et al., 2005a; Legocka and Kluk, 2005; Liu et al., 2006) under salinity. Transcript levels of other polyamine biosynthesis-related genes are also increased under salt stress, e.g., *SAMDC* in rice (Li and Chen, 2000b), soybean (Tian et al., 2004), wheat (Li and Chen, 2000a), *Arabidopsis* (Urano et al., 2003) and apple (Hao et al., 2005b), *SPD* and *SPM* in *Arabidopsis* (Urano et al., 2003), and maize (Rodríguez-Kessler et al., 2006). Tolerance to drought was improved by constitutive over-expression of oat *ADC* in rice (Capell et al., 1998), with a simultaneous effect on plant development. When polyamine accumulation was induced by over-expression of oat *ADC* or *Tritodermum SAMDC* under the control of an ABA-inducible promoter, rice plants were more resistant to high salinity (Roy and Wu, 2001, 2002). Furthermore, over-expression of the *Datura stramonium ADC* gene under the control of the stress activated maize ubiqitin-1 promoter conferred tolerance to osmotic stress in rice (Capell et al., 2004). For a broader overview of transgenic approaches; see Gill and Tuteja (2010) and Marco et al. (2012).

Here we investigated changes in polyamine content and expression levels of all genes encoding enzymes involved in polyamine biosynthesis in a wide range of rice cultivars under long-term moderate salt stress using two different NaCl concentrations and we explored the possible correlations between physiological parameters, polyamine content, and gene expression levels and salt sensitivity of those 18 rice cultivars. In addition, by comparing polyamine levels and changes in gene expression with results obtained under mild drought stress conditions, we were able to classify the different genes into either salt- or drought- or generally stress-responsive.

## Materials and methods

### Plant material, cultivation, and salt stress treatment

Eighteen rice (*Oryza sativa* L.) cultivars originating either from the IBT (Institute of Biotechnology, Hanoi, Vietnam) or from the IRRI (International Rice Research Institute, Manila, Philippines) {Nipponbare (IRGC accession 12731) (NB), Taipei 309 (IRGC accession 42576) (TP), IR57311-95-2-3 [IRGC accession 17509 (INGER)] (IR) and Zhonghua} were grown under control and salt stress conditions in three independent experiments in a climate chamber. For a complete list of cultivars see Table 1. The design was a randomized complete block design with five blocks, each containing one hydroponic culture box with 0, 50, and 100 mM NaCl, respectively. Boxes were randomized within the blocks.

**Table 1:** **List of cultivars of *Oryza sativa* L. used for salt stress experiments**.

**Cultivar**	**Number**	**Subspecies**	**Origin**
CR203	1	Indica^*^	IBT
DR2	2	Indica^*^	IBT
C70	4	Indica^*^	IBT
C71	5	Indica^*^	IBT
Doc Do	7	*Indica*	IBT
Doc Phung	8	*Indica*	IBT
Cuom	14	Indica/japonica^*^	IBT
Nuoc Man	21	*Indica*	IBT
Lua Man	22	Indica^*^	IBT
Nep Man	23	Indica^*^	IBT
Nuoc Man 1	25	*Indica*	IBT
Cham	26	*Indica*	IBT
Cham Bien	27	*Indica*	IBT
Cha Va	28	*Indica*	IBT
Nipponbare	50	*Japonica*	IRRI
Taipei 309	51	*Japonica*	IRRI
IR57311-95-2-3	52	*Indica*	IRRI
Zhonghua	53	*Japonica*	IRRI

*For genotyping of the Vietnamese cultivars marked with an asterisk, see Degenkolbe et al. (2013). IBT, Institute of Biotechnology, Hanoi, Vietnam; IRRI, International Rice Research Institute, Manila, Philippines*.

Seeds were germinated at 28°C for 10 days. Plantlets were transferred to a climate chamber with 12 h light phase at a photon flux density of 600 μE m^−2^ s^−1^ (Lamps: Iwasaki Eye MT 400 DL/BH E40, DHL Licht, Wülfrath, Germany); temperature was 26°C (day) and 22°C (night), with a relative humidity of 70%. Plants were grown hydroponically in 10 l polypropylene boxes filled with medium according to Yang et al. (1994) and covered with a lid. Plantlets were fixed to holes in the lid with a piece of foam material and covered with a transparent lid for 2 days. After 14 days the growth medium was exchanged for medium with the appropriate salt concentration (0, 50, 100 mM NaCl), which was renewed after an additional week.

After 14 days of salt stress treatment, plants were sampled 4–6 h after the beginning of the light period. The middle sections of leaves were selected for physiological measurements to avoid taking material from the elongation zone at the base of the leaf blade or senescent tissue at the top of the leaves, especially in stressed plants. Fully expanded green leaf blades were harvested and immediately frozen in liquid nitrogen for transcript and polyamine analysis. The remaining plant was harvested to determine shoot fresh (FW) and dry weight (DW, 48 h, 80°C).

For a detailed description of the drought stress experiments please refer to Do et al. (2013).

21 rice cultivars were grown under control and drought conditions in controlled climate chambers in 12 h days (600 μE m^−2^ s^−1^) at 26°C and 75% relative humidity and 12 h nights at 22°C and 70% relative humidity. Plants were grown in 540 g sand mixed with 8 g of Lewatit HD 50 (Lanxess, Langenfeld, Germany) and 0.4 g Fetrilon Combi (Compo, Münster, Germany). Pots were positioned. Rice plants were grown in boxes filled with water to the level of the substrate surface. 26 days after sowing drought stress was applied by water removal from half of the boxes. When the permanent wilting point for 50% of the plants was reached soil water content was kept constant for 14 days by weighing each pot at the end of the light period and adding the amount of water lost during the last 24 h.

### Physiological characterization of the plants and sampling

The leaf phenotype of stressed and control plants was visually assessed for individual plants before and during salt stress treatment 24 DAS (before stress treatment), 30 DAS (6 days after stress treatment), and 37 DAS (13 days after stress treatment) based on the stress damage score of the IRRI (Mitchell et al., 1998). An average stress damage score considering chlorosis and necrosis and using a scale from 1 to 9 was given to every plant with “1” representing plants with undamaged leaves, “9” almost or completely dead plants. The number of tillers was counted, and plant height (Wopereis et al., 1996) measured at the same time.

Chlorophyll-*a* fluorescence and leaf temperature were measured with a pulse-amplitude-modulated Dual- PAM-100 fluorometer (WALZ, Effeltrich, Germany) on the middle section of the second fully expanded leaf during mid-day without dark adaptation and under climate chamber conditions. The effective quantum yield of PS II (ΔF/Fm′) was determined from the maximum light-adapted fluorescence yield (Fm′) and the current fluorescence yield (Ft) as [ΔF/Fm′ = (Fm′ - Ft)/Fm′].

Salt score values were ranked and the average rank calculated within each experiment. Results of three experiments were combined and mean and standard error of the average ranks was calculated for every cultivar. The relative DW of the shoot was calculated by dividing the average shoot DW under salt condition by the respective control value for each cultivar and experiment.

A description of the physiological characterization and the ranking of cultivars under drought stress conditions are given in Do et al. (2013).

### Polyamine analysis

Free polyamines (Put, Spd, and Spm) were quantified by High Performance Liquid Chromatography (HPLC) as described in Do et al. (2013). Data were analyzed using the Chromeleon software (Dionex, Germering, Germany) and calibration curves obtained from the pure substances. Detailed data on polyamines levels under drought conditions are given in Do et al. (2013).

### Quantitative RT-PCR (qRT-PCR)

qRT-PCR was performed with the ABI Prism 7900HT (Applied Biosystems, Foster City, CA) as described in Do et al. (2013). Primers were designed using PrimerExpress (Version 2.0, Applied Biosystems) and all primer sequences are given in Do et al. (2013). Data were analyzed using the SDS 2.0 software (Applied Biosystems) and normalized based on the expression of the housekeeping genes actin 1 and cyclophilin. Normalized gene expression was calculated by dividing the average relative expression (primer efficiency P to the power of cycle number Ct) of the two housekeeping genes (H1 and H2) by the relative expression of the gene of interest (GOI) (Degenkolbe et al., 2009). Primer efficiency was calculated using LinRegPCR (Ramakers et al., 2003). Fold change was calculated as log_2_ of the ratio of relative expression of genes under stress conditions to relative expression under control conditions. Detailed gene expression data of drought-tolerant and drought-sensitive cultivars are given in Do et al. (2013).

### Statistics

Spearman correlations between the mean rank of the salt stress damage score and the respective means of all performance parameters across three experiments were determined using the rcorr function provided by the R-package “Hmisc” after a correction for outliers. The significance of differences between treatments was analyzed using unpaired, two-sided *t*-tests in Microsoft Excel. Significance levels in the figures are indicated as: 0.05 > *p* > 0.01 (^*^), 0.01 > *p* > 0.001 (^**^), *p* < 0.001 (^***^).

## Results

### Phenotyping of rice cultivars under salt conditions

**Figure 1:**
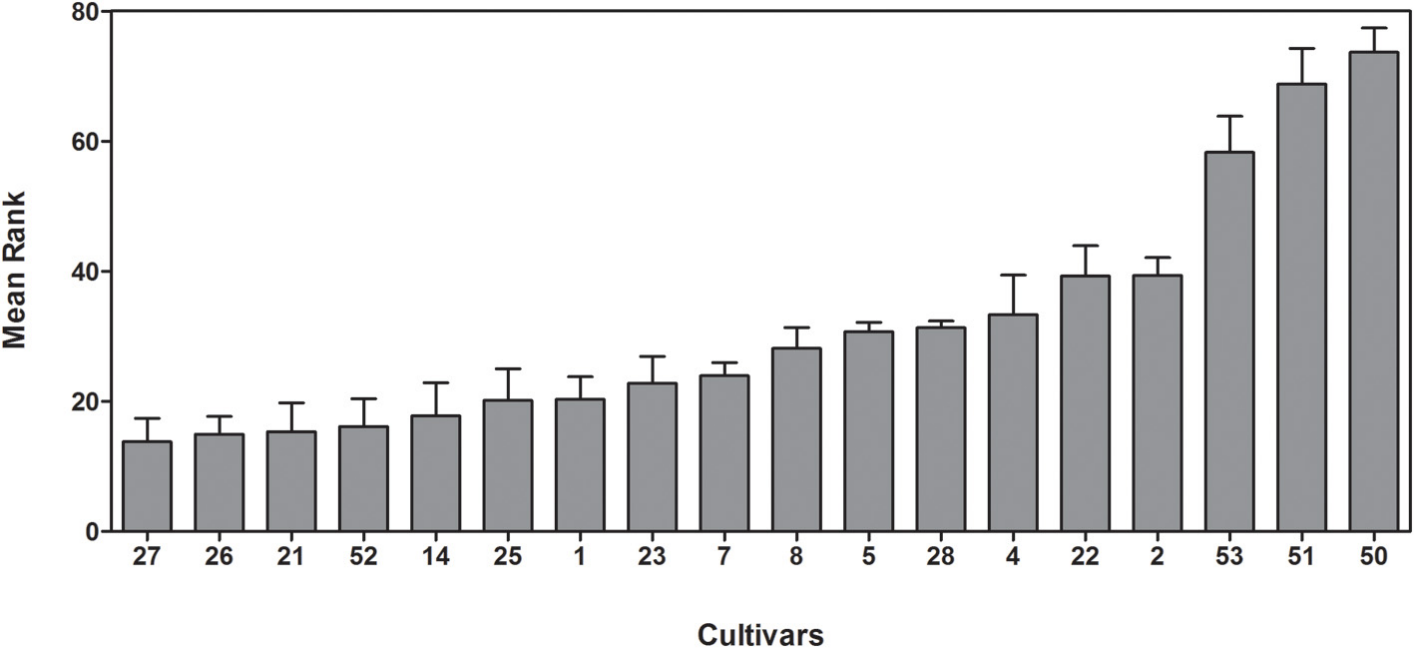
**Classification of 18 rice cultivars after 13 days at 100 mM NaCl in hydroponic culture**. Scores were ranked and the average rank of three independent experiments is shown with standard errors. Cultivars are sorted from the most tolerant to the most sensitive from left to right.

18 rice cultivars representing either *japonica* or *indica* subspecies, including cultivars from a Vietnamese breeding program and four well characterized IRRI cultivars (Table 1), were subjected to two different salt stress conditions, 50 and 100 mM NaCl, in the vegetative stage using hydroponic culture in controlled growth chambers. Before salt treatment and after 6 and 13 days of salt stress plants were characterized and compared to control plants by scoring their leaf phenotype and by measuring plant height, tiller number, FW, and DW. Differences between cultivars were highest after 14 days at 100 mM NaCl. At this time point cultivars could be clearly classified based on their salt sensitivity using the average rank of visual scoring (Figure 1). Mean ranks from 13.8 to 73.7 indicated a large natural variation for salt tolerance between the selected cultivars. The *indica* cultivars Cham bien (27), Cham (26), and Nuoc man (21) were the most tolerant with scoring values from 13.8 to 14.9, whereas the *japonica* cultivars Nipponbare (50), Taipei 309 (51), and Zhonghua (53) were the most sensitive, with scoring values ranging from 58.3 to 73.7. All cultivars of the *japonica* subspecies could be classified as sensitive. The most sensitive *indica* cultivars were C70 (4), DR2 (2), and Lua Man (22).

**Figure 2:**
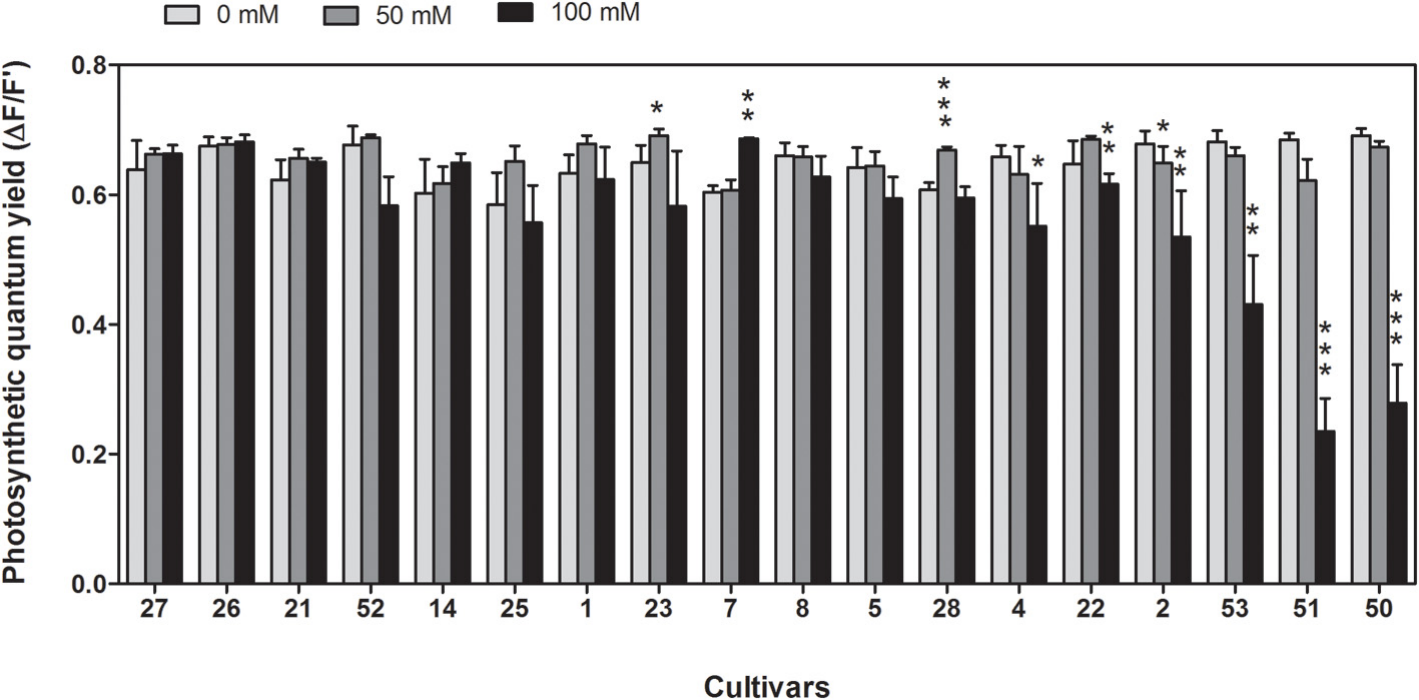
**Photosynthetic quantum yield of 18 rice cultivars under control conditions and after 13 days at 50 mM or 100 mM NaCl in hydroponic culture**. Means of three independent experiments with five replicate plants each are shown with standard errors. Significance levels are indicated as: *p* < 0.001 (^***^), 0.001 < *p* < 0.01 (^**^), 0.01 < *p* < 0.05 (^*^) in comparison to control. Cultivars are sorted from the most tolerant to the most sensitive from left to right.

Integrity of the photosynthetic machinery was investigated by measuring the effective quantum yield of photosynthesis after 13 days of stress for both NaCl concentrations (Figure 2). While in most cultivars quantum yield was not impaired by salt treatment, a significant reduction occurred at 100 mM NaCl in the six most sensitive cultivars, including both *indica* and *japonica* subspecies. At mild salt stress conditions of 50 mM NaCl no significant reduction of effective quantum yield was observed.

**Figure 3:**
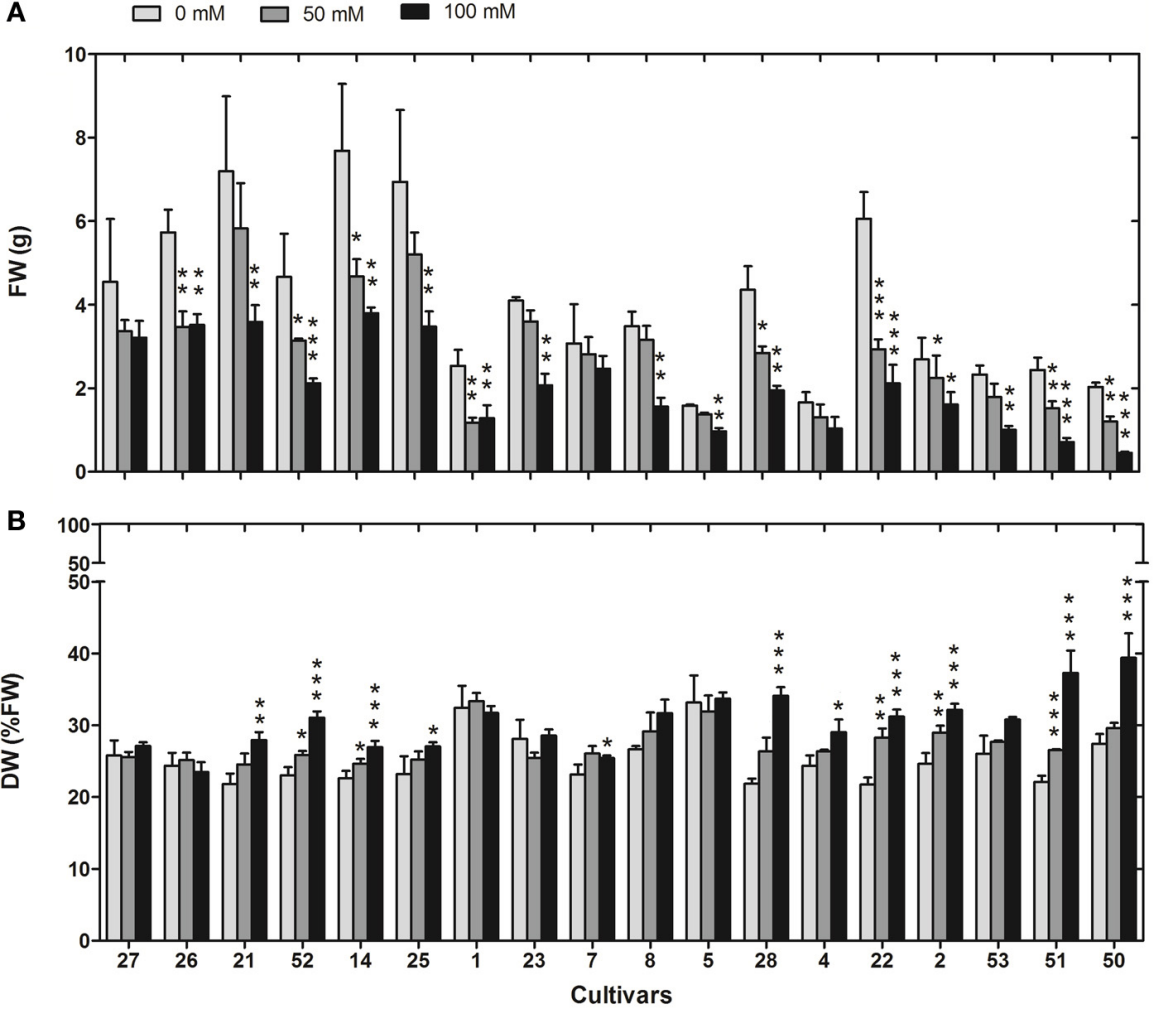
**FW (A) and DW as %FW (B) of 18 rice cultivars under control conditions and after 13 days at 50 mM or 100 mM NaCl in hydroponic culture**. Means of three independent experiments with five replicate plants each are shown with standard errors. Significance levels are indicated as: *p* < 0.001 (^***^), 0.001 < *p* < 0.01 (^**^), 0.01 < *p* < 0.05 (^*^) in comparison to control. Cultivars are sorted from the most tolerant to the most sensitive from left to right.

Plant height was cultivar dependent under control conditions with largest plants in cultivar 25 and smallest in cultivar 4. A reduction due to 100 mM NaCl treatment occurred in four cultivars (14, 8, 22, 50) and showed no tolerance dependent pattern (not shown). Tiller number under control conditions was also highly cultivar dependent with the highest number in cultivar 22 and the lowest in 51, and it was reduced by 14 to 41% across cultivars under salt stress (data not shown). FW was significantly affected by salt treatments of 50 and 100 mM NaCl in most cultivars (Figure 3A). In the most sensitive cultivar Nipponbare (50) it was reduced to 22% of the control value. Strikingly, relative DW as %FW was increased at 100 mM NaCl with descending tolerance between no change in the most tolerant and an increase of 39% in the most sensitive cultivar (Figure 3B). The most tolerant cultivars showed no significant change of relative DW under salt stress. Spearman correlation analysis for the sensitivity rank at 100 mM NaCl and the ratio of all available growth parameters in comparison to control conditions revealed significant negative correlations between sensitivity rank and photosynthetic quantum yield at 50 and 100 mM NaCl and FW at 100 mM NaCl, and a significant positive correlation with the relative DW at 100 mM NaCl (Table 2).

**Table 2:** **Spearman correlation analysis between the mean sensitivity rank at 100 mM NaCl and the ratio of all available growth parameters under stress compared to control conditions, absolute polyamine content in nmol g^−1^ DW and ratio of polyamine content under stress in comparison to control conditions**.

**Parameter (Absolute content or ratio to control)**	***r***	***p*-value**
Ratio photosynthetic yield 50 mM	−0.589	*0.0101*
Ratio photosynthetic yield 100 mM	−0.736	**0.0005**
Ratio tiller number 50 mM	0.059	0.8167
Ratio tiller number 50 mM	−0.143	0.5701
Ratio plant height 50 mM	−0.032	0.8997
Ratio plant height 100 mM	−0.257	0.3033
Ratio FW 50 mM	−0.346	0.1600
Ratio FW 100 mM	−0.585	*0.0107*
Ratio DW 50 mM	0.422	0.0810
Ratio DW 100 mM	0.587	*0.0104*
Put 0 mM	0.820	**<0.0001**
Spd 0 mM	0.150	0.5534
Spm 0 mM	−0.276	0.2684
Put 50 mM	0.298	0.2293
Spd 50 mM	0.069	0.7851
Spm 50 mM	−0.013	0.9579
Put 100 mM	−0.575	*0.0126*
Spd 100 mM	−0.560	*0.0156*
Spm 100 mM	−0.422	0.0810
Ratio Put 50 mM	−0.800	**0.0001**
Ratio Put 100 mM	−0.843	**<0.0001**
Ratio Spd 50 mM	−0.061	0.8103
Ratio Spd 100 mM	−0.676	***0.0021***
Ratio Spm 50 mM	0.179	0.4784
Ratio Spm 100 mM	−0.094	0.7109

*P-values below 0.05 are highlighted in italic, below 0.01 highlighted in boldface and italic, and below 0.001 highlighted in boldface numbers*.

### Polyamine content of rice changes during salt stress

**Figure 4:**
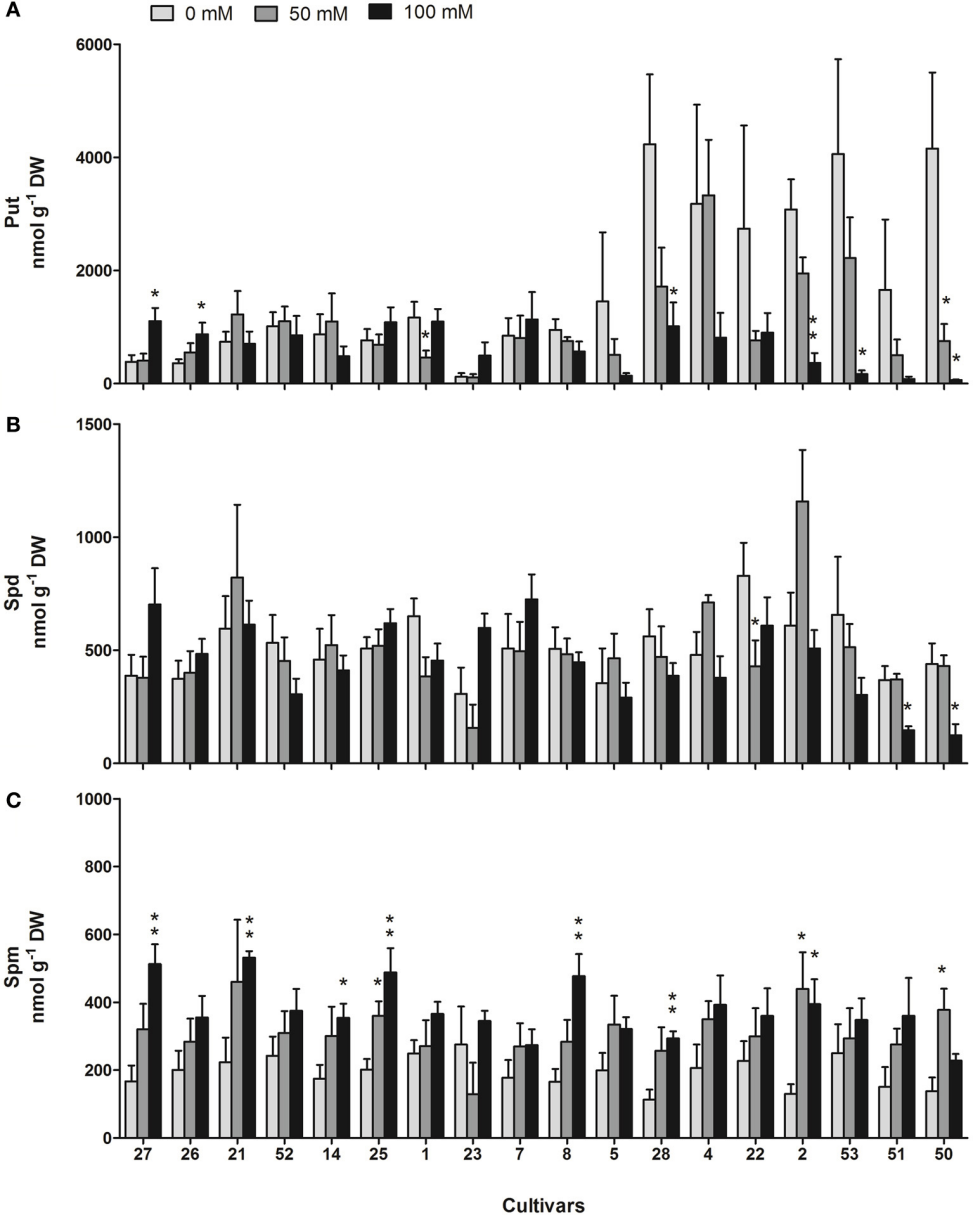
**Polyamine content under control and salt stress (50 and 100 mM NaCl) conditions**. The panels show Put **(A)**, Spd **(B)**, and Spm **(C)** content in leaves of 18 rice cultivars. Each value represents the mean (±s.e.m.) of one experiments with five replicate plants each. Significance levels are indicated as: 0.001 < *p* < 0.01 (^**^), 0.01 < *p* < 0.05 (^*^) in comparison to control. Cultivars are sorted from the most tolerant to the most sensitive from left to right.

Pool sizes of predominant free polyamines (Put, Spd, Spm) were measured in leaves of all cultivars after 14 days of salt stress in comparison to the control. Figure 4 shows the respective values in all investigated cultivars sorted by their tolerance. Put showed the highest genotypic variation, especially under control conditions, with values ranging from 120 to 4230 nmol g^−1^ DW. Under control conditions, Put content was significantly higher in the more sensitive cultivars including *indica* and *japonica* ssp. compared to more tolerant ones. For the eight most sensitive cultivars, Put values under control conditions ranged from 1450 to 4230 nmol g^−1^ DW, whereas values in the eight most tolerant cultivars reached only values from 120 to 1166 nmol g^−1^ DW. After 14 days of salt treatment a strong decrease of Put at both NaCl levels was restricted to the eight most sensitive cultivars with 11 and 1.5% of the control values for cultivars 2 and 50 at 100 mM NaCl, respectively. All other cultivars showed either an increase (26, 27) or no significant change. The ratio of Put at 50 and 100 mM NaCl in comparison to control conditions was negatively correlated with the salt sensitivity score, confirming a stronger relative decrease of Put content in sensitive as compared to tolerant cultivars (Table 2). Strikingly, the most sensitive cultivars contained the highest absolute amounts of Put under control conditions, indicated by a positive correlation of Put content with the salt sensitivity score (*r* = 0.82, *p* = 0.00003).

In contrast to Put, Spd content was not changed under salinity, except for a significant reduction at 100 mM NaCl in the most sensitive cultivars 51 and 50 (to 40 and 28%, respectively) and an increase at 100 mM NaCl in the most tolerant cultivar 27 (to 181%) that was, however, not significant (Figure 4). Surprisingly, absolute Spd levels at 100 mM NaCl showed a significant negative correlation (*r* = −0.56, *p* = 0.0155) with salt sensitivity and also the ratio of Spd at 100 mM NaCl in comparison to control was negatively correlated with salt sensitivity (Table 2).

General increases of Spm levels were observed in all cultivars under both salt stress conditions with up to 3-fold increases at 100 mM NaCl. However, the predominant compound in most cultivars under salinity conditions (100 mM) was still Put followed by Spd and Spm with the exception of the three most sensitive cultivars 53, 51, and 50 with a higher Spm content, followed by Spd and Put.

### Expression analysis of polyamine biosynthesis genes

**Figure 5:**
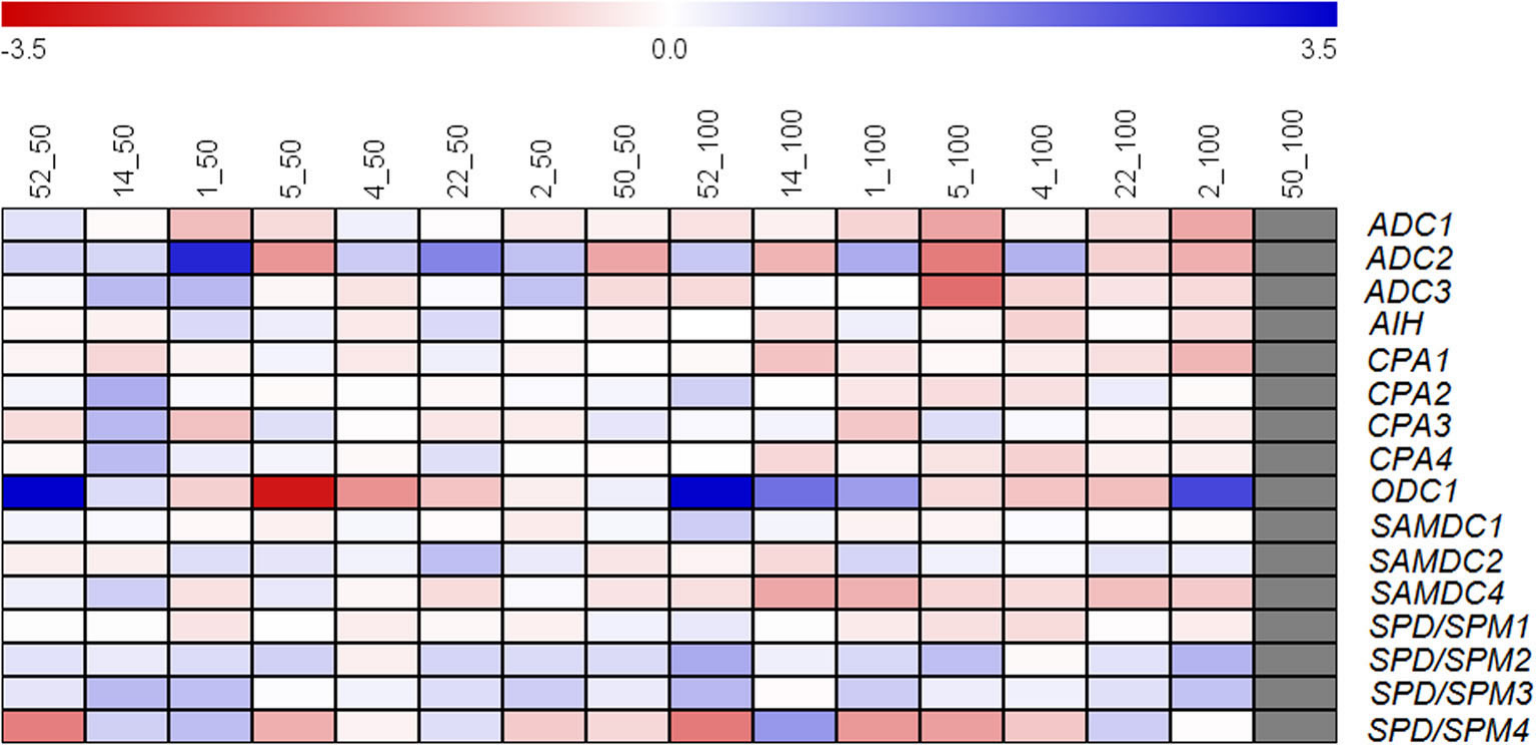
**Log_2_ fold change of expression of genes, encoding enzymes involved in polyamine biosynthesis, under salt stress (50, 100 mM) as compared to control conditions in eight rice cultivars differing in salt tolerance**. Leaves from five different plants were pooled for RNA extraction. Each value represents the mean of three technical replicates. Cultivars are sorted from the most tolerant to the most sensitive from left to right.

Expression of 16 genes encoding enzymes involved in polyamine biosynthesis was analyzed in leaves of eight cultivars after 14 days of salt treatment (50 and 100 mM NaCl) using qRT-PCR. The eight cultivars were selected based on their widely differing salt tolerance and included both subspecies with cultivar 52, 14, and 1 belonging to the more tolerant, 5, 4, 22, 2, and 50 to the more and most sensitive ones. The log_2_ fold change between gene expression under salt compared to control conditions is shown in Figure 5. In general, increased gene expression is more obvious at 50 mM than at 100 mM NaCl with the exception of *ODC1* in some cultivars and *SPD/SPM2* and *SPD/SPM3* in all cultivars. At 50 mM NaCl *ADC2*, a gene involved in the synthesis of Put, was induced in almost all cultivars except cultivar 5 and the most sensitive cultivar 50. Also *SPD/SPM2* and *SPD/SPM3* were induced in almost all cultivars under this condition. There are two alternative pathways to synthesize Put, either indirectly from arginine, catalyzed by the enzymes ADC, AIH, and CPA, or directly from ornithine, catalyzed by ODC. In two of the tolerant cultivars, genes encoding enzymes for both pathways (*ADC2, ODC1*) were induced by salt stress, whereas in sensitive cultivars the expression of only one of these genes, either *ADC2* or *ODC1* was increased.

The fold change in the expression of genes encoding enzymes involved in the following steps of the polyamine biosynthesis pathway, leading to the synthesis of Spd and Spm, varied for cultivars and genes with *SAMDC2* being induced in most of the cultivars at both salt levels. Additionally, *SPD/SPM2* and *SPD/SPM3* were induced in all cultivars under both conditions. No correlations could be found between the log_2_ fold change of gene expression and the salt sensitivity of the cultivars.

### Comparison of the salt and drought responses of polyamine metabolism and gene expression

For comparison of the responses of rice to salt and drought stress at the level of polyamine metabolism, data from three salt-tolerant (52, 14, 1) and three salt-sensitive cultivars (22, 2, 50) were summarized and averaged. These cultivars were chosen to allow comparison of their responses to drought stress investigated in a previous study (Do et al., 2013). Do and co-workers analyzed drought tolerance and the response of polyamine metabolism in 21 rice cultivars with an overlap of nine cultivars between the two studies. For the comparison, three drought-sensitive cultivars (22, 2, 50) and three drought-tolerant cultivars (1, 4, 52) were selected. Cultivar 14 in our selection of salt-tolerant cultivars, had to be replaced by 4 in the selection of drought-tolerant cultivars, because cultivar 14 was not drought-tolerant.

**Figure 6:**
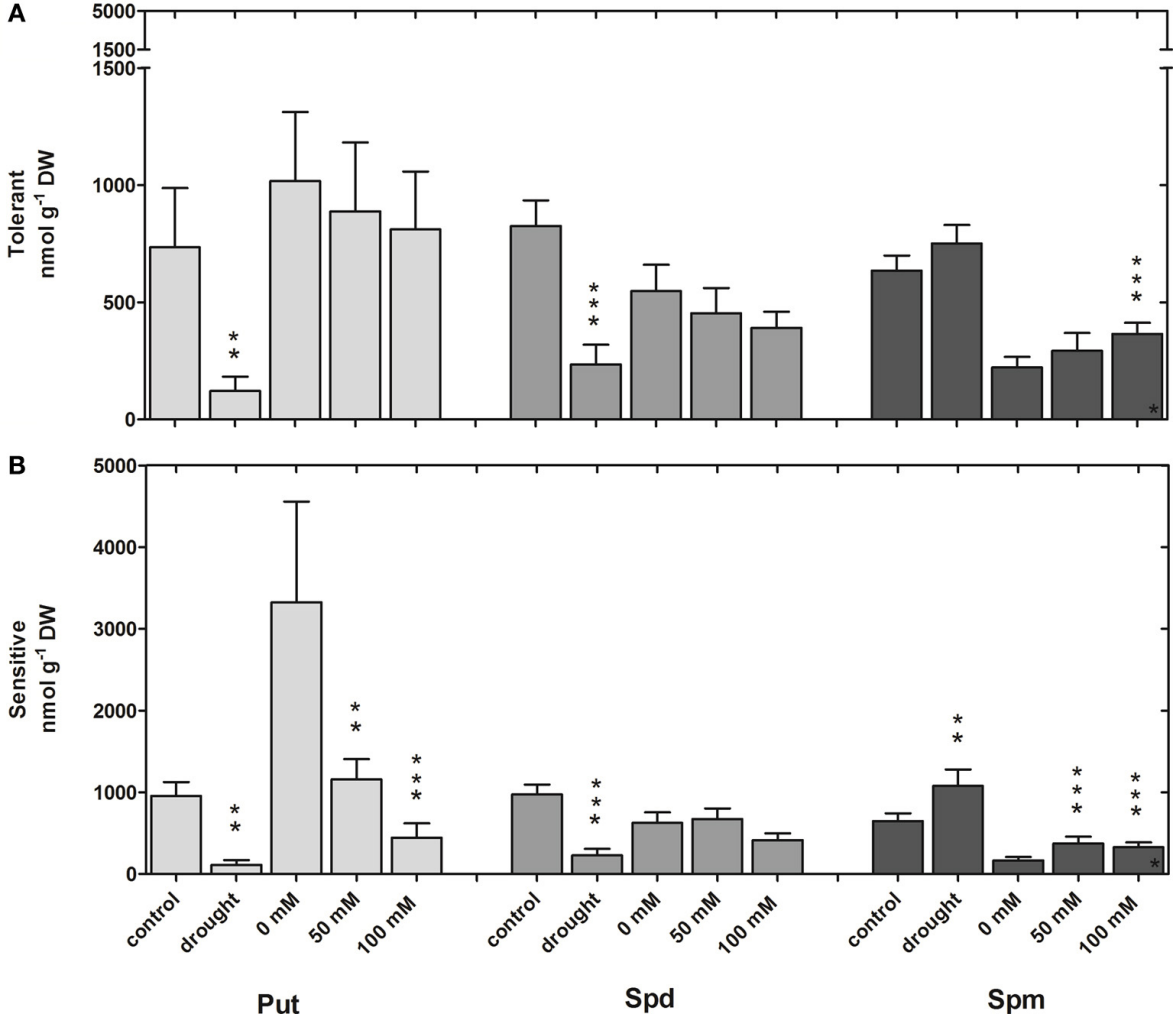
**Comparison of polyamine contents under drought and salinity stress**. Put, Spd, and Spm contents are shown for drought- and salt-tolerant (52, 4, 1) **(A)** and drought- (22, 2, 50) and salt-sensitive cultivars (22, 2, 50) **(B)** under control and stress conditions. Each value represents the mean (±s.e.m.) of three cultivars with five replicate plants each. Significance levels are indicated as: *p* < 0.001 (^***^), 0.001 < *p* < 0.01 (^**^), 0.01 < *p* < 0.05 (^*^) in comparison to the respective control.

Average polyamine levels (Put, Spd, Spm) of the three tolerant and sensitive cultivars were compared under drought and salt (50, 100 mM NaCl) conditions (Figure 6). Under control conditions Put levels were similar for the different growth conditions (growth in sand for drought and hydroponic culture for salt stress) with values between 100 and 800 nmol g^−1^ DW except for sensitive cultivars in hydroponic culture with over 4150 nmol Put g^−1^ DW. Put levels were clearly reduced under both drought and salt conditions with the exception of tolerant cultivars at 50 and 100 mM NaCl. Spd values were slightly lower in hydroponic culture compared to plants grown in sand under control conditions. While Spd was significantly reduced after 18 days of drought stress in all cultivars, no significant changes were observed under salt stress. Spm levels were also similar for both growth conditions in the absence of stress and were significantly elevated in sensitive cultivars under drought and both salt treatments, while they rose only at 100 mM NaCl in the tolerant cultivars.

In summary, tolerant cultivars are able to keep their Put levels constant under salt stress and increase their Spm levels only slightly at the high salt concentration, whereas in sensitive cultivars Put levels are strongly decreased and Spm levels increased. Under drought conditions, polyamine levels show the same pattern in sensitive cultivars with reduced Put and increased Spm levels. In contrast, the pattern in drought tolerant cultivars under drought is different from the salt response with strongly decreased Put and Spd levels and no changes of Spm.

**Figure 7:**
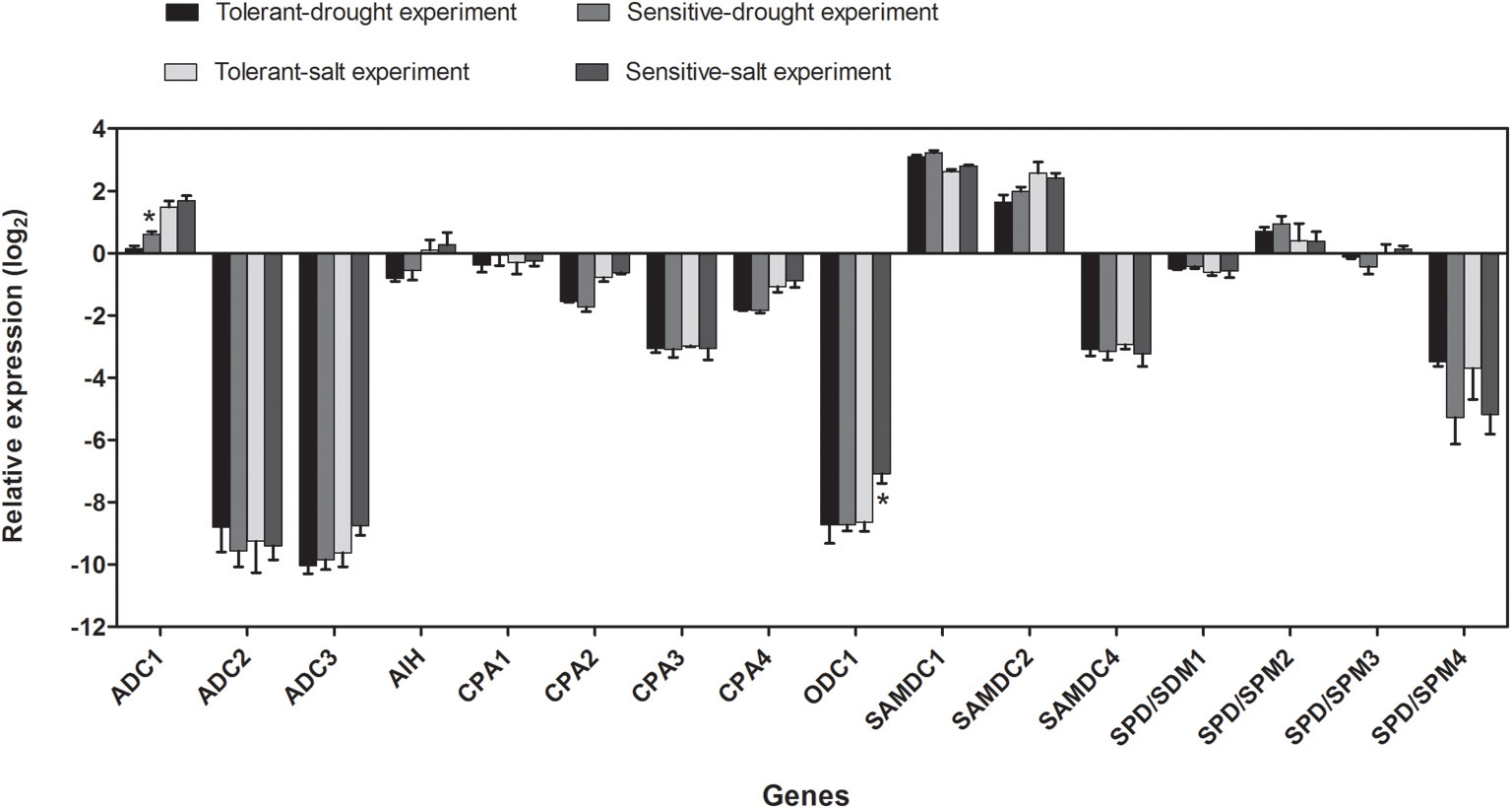
**Relative average expression (log_2_) of genes encoding enzymes involved in polyamine biosynthesis under control conditions in three tolerant vs. sensitive cultivars used in drought and salt stress experiments**. Relative gene expression (log_2_) in comparison to the housekeeping genes is shown for drought- and salt-tolerant (52, 4, 1) and drought- (22, 2, 50) and salt-sensitive cultivars (22, 2, 50) under control conditions. Data represent the means of three cultivars from either one or two biological replicates with three technical replicates each. Significance levels are indicated as: 0.01 < *p* < 0.05 (^*^) in comparison to the respective tolerant cultivars.

To compare gene expression levels under different culture conditions, we calculated the average gene expression of the three tolerant and sensitive cultivars, respectively. A positive value of relative expression (log_2_) represents a higher expression of a GOI in comparison to the housekeeping genes and a negative value a lower expression. Differences in gene expression between growth conditions could be observed for *ADC1*, with a lower expression in sand-grown plants compared to plants grown hydroponically, and *ODC1*, showing a higher expression in salt-sensitive cultivars grown in hydroponic culture (Figure 7).

**Figure 8:**
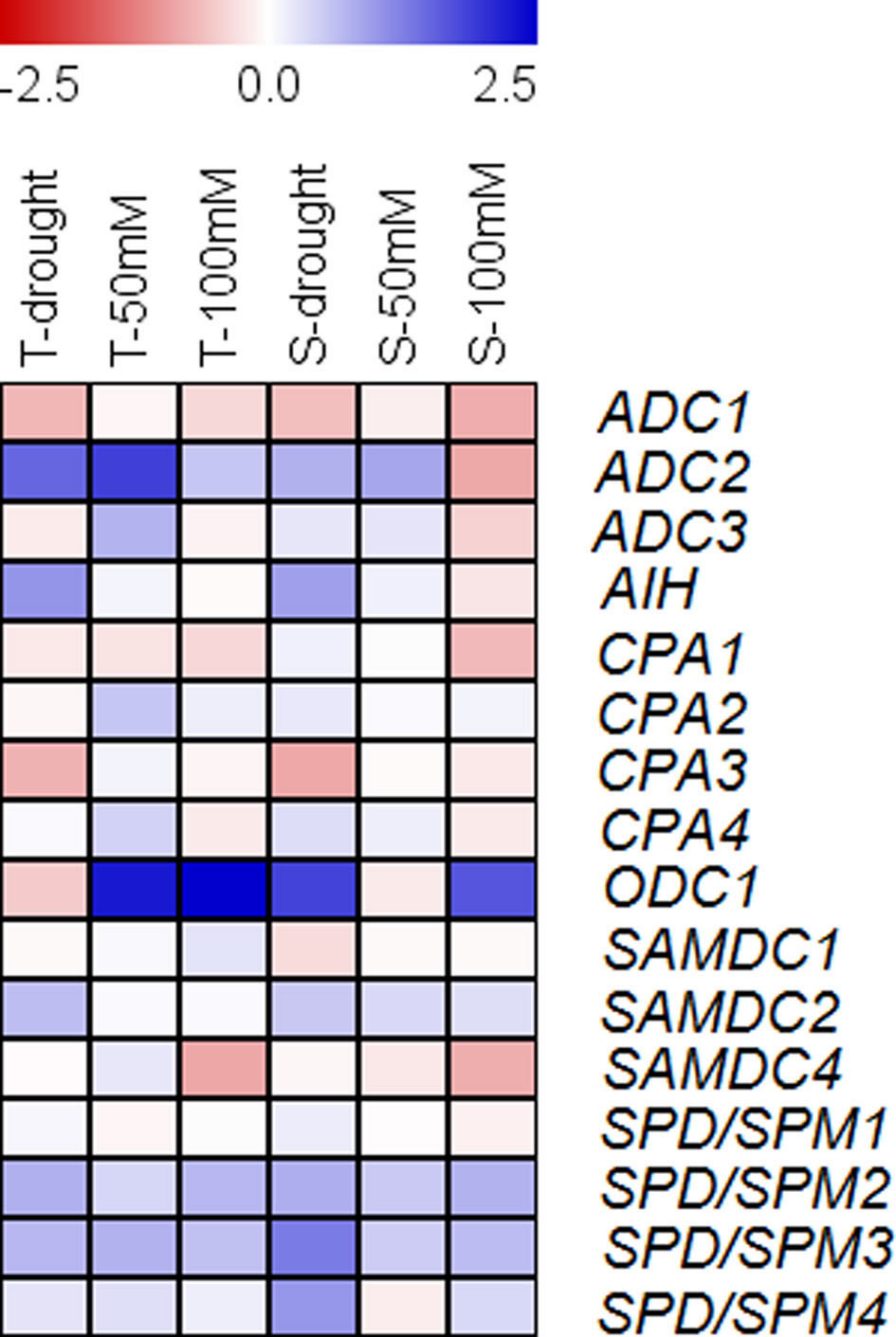
**Log_2_ fold change of expression of genes, encoding enzymes involved in polyamine biosynthesis, under drought and salt conditions as compared to control in three tolerant vs. sensitive cultivars of the respective stress condition**. Log_2_ fold change of gene expression is shown for drought- and salt-tolerant (T) (52, 4, 1) and drought- (22, 2, 50) and salt-sensitive cultivars (S) (22, 2, 50). Data represent the means of three cultivars from either one or two biological replicates with two technical replicates each.

Additionally, log_2_ fold change values of average gene expression of the selected tolerant and sensitive cultivars under both stresses were calculated (Figure 8). At drought and salt conditions compared to control *ADC1* expression was reduced in both groups with the highest reduction in sensitive cultivars at 100 mM NaCl, while *ADC2* was highly induced under stress with the exception of sensitive cultivars at 100 mM NaCl. Log_2_ fold change of gene expression of *ADC3* showed a fluctuating pattern with the strongest reduction again in sensitive cultivars at 100 mM NaCl. This seemed to be partly compensated by a high induction of *ODC1*, which catalyzes an alternative pathway of Put synthesis. On the other hand, *ODC1* is also induced under all other conditions except for drought in tolerant and at 50 mM NaCl in sensitive cultivars. Whereas *SAMDC2* was always induced in sensitive cultivars under all conditions, this induction was only observed under drought in tolerant cultivars. Log_2_ fold change of gene expression of *SAMDC4* showed a clear stress specific pattern, whereas *SPD/SPM2, SPD/SPM3*, and *SPD/SPM4* (with one exception) were generally stress induced in both tolerance groups.

Based on expression profiles of cultivars differing in tolerance, the response in gene expression after drought and salt stress could be differentiated for 13 genes. The investigated genes were divided into generally stress-induced genes *(ADC2, ODC1, SPD/SPM2, SPD/SPM3*) with two genes showing tolerance related differences under salt (*ADC2*) or drought conditions (*ODC1)*, one generally stress-repressed gene *(ADC1*), constitutively expressed genes *(ADC3, CPA1, CPA2, CPA4, SAMDC1, SPD/SPM1*), one specifically drought-induced gene *(AIH*), one specifically drought-repressed gene *(CPA3*), and one specifically salt-stress repressed gene *(SAMDC4*) revealing both overlapping and specific stress responses.

## Discussion

Despite several published studies on the response of polyamine metabolism to salt stress in rice, comparative analyses of a large number of cultivars are rare. For the present investigation of the polyamine response in 18 cultivars, a detailed physiological characterization at two different salt concentrations in an early vegetative stage was performed and revealed a large variation of salt tolerance among the cultivars based on a rank of scoring data that indicated a higher salt sensitivity of *japonica* as compared to *indica* cultivars. It was previously reported that salt tolerance of *indica* cultivars was higher than that of *japonica* cultivars, denoted by a lower reduction of growth and a better Na^+^ exclusion (Lee et al., 2003). Among the cultivars investigated in the present study, Nipponbare was known to be salt-sensitive (Karan et al., 2012), while for all other cultivars no salt tolerance classification was available. Our phenotypic ranking was supported by a decrease of photosynthetic quantum yield and a reduction of FW under salt stress, especially in sensitive cultivars. Decreased photosynthetic yield is well known from previous studies (Lutts et al., 1996; Yamamoto et al., 2004) and the lack of an effect in a salt tolerant cultivar (Pokkali) was also shown (Dionisio-Sese and Tobita, 2000). Reduced FW under salinity was also described by Su and Wu (2004), who used this parameter for the classification of salt tolerance. The three most sensitive cultivars, 50, 51, and 53, all *japonica* ssp., as well as the two most tolerant cultivars, 26 and 27 (*indica*), were also clustered according to their metabolomic profiles considering changes of four metabolite pools under salinity suggesting a similar metabolic state (Zuther et al., 2007).

### Effects of salinity stress on free polyamine contents in leaves

A general increase of Spm was observed under salinity conditions for all cultivars in the present study, which is in agreement with previous results obtained in rice (Krishnamurthy and Bhagwat, 1989; Maiale et al., 2004) and other species (Sanchez et al., 2005). Spm may be involved in the stabilization of membranes (Tassoni et al., 1998; Rajasekaran and Blake, 1999) and nucleic acids (Hultgren and Rau, 2004), scavenging of free radicals (Lester, 2000), osmotic adjustment (Aziz et al., 1999), regulation of ion nutrition (Chattopadhyay et al., 2002), and regulation of senescence (Lahiri et al., 2004). It can improve the viability of protoplasts (Tiburcio et al., 1986) and participates in the prevention of electrolyte leakage and chlorophyll loss (Chattopadhyay et al., 2002) as well as enhanced stem elongation growth (Rajasekaran and Blake, 1999). Polyamines are also able to block ion channels thereby reducing NaCl-induced K^+^ efflux proportional to their charge (Zhao et al., 2007). Furthermore Spm can provoke a net Ca^2+^-efflux which might influence ROS and PA signaling (Pottosin et al., 2012).

These multiple functions suggest a beneficial role for Spm under stress conditons, independent of the tolerance level. Spm was also identified as the polyamine responsible for salt acclimation in *Arabidopsis* using transgenic lines overexpressing oat ADC (Alet et al., 2011b). Furthermore it was shown that *Arabidopsis* mutants, defective in the synthesis of Spm (*spms-1*), accumulated more Na^+^ and were impaired in survival experiments compared to control (Alet et al., 2012).

Despite only slight changes of Spd content in most of the cultivars under salinity, a significant negative correlation of Spd levels and of the changes under salt stress with salt sensitivity was found. This is in agreement with findings of Krishnamurthy and Bhagwat (1989) who reported that salt-tolerant rice accumulates high levels of Spd and Spm. Over-expression of *SPD* from *Cucurbita ficifolia* in *Arabidopsis* results in significantly increased Spd content in leaves and in enhanced tolerance to various abiotic stresses (Kasukabe et al., 2004). Spd treatment also induced recovery from salinity-induced damage of the plasma membrane and PM-bound H^+^-ATPase in salt-tolerant as well as salt-sensitive cultivars (Roy et al., 2005).

Strikingly, polyamine levels changed differently in tolerant and sensitive cultivars, with higher levels of Put under control conditions and a stronger decrease under salt stress in sensitive cultivars. This resulted in a shift of the predominant polyamine at 100 mM NaCl from Put in tolerant to Spm in the three most sensitive cultivars. These results may help to explain contradictory findings for changes of Put levels in the literature. It was earlier suggested that endogenous levels of Put might be limiting for salt resistance (Gupta et al., 2013). Our results are in agreement with Maiale et al. (2004), who also found a larger decrease of Put in sensitive as compared to tolerant cultivars. However, it has also been reported that Put strongly accumulates in response to osmotic (Flores and Galston, 1982; Aziz and Larher, 1995; Liu et al., 2004) and salinity stress (Basu et al., 1988; Krishnamurthy and Bhagwat, 1989; Katiyar and Dubey, 1990; Lefèvre et al., 2001). This accumulation was considered to be protective, conferring a selective advantage to the stressed plants. Put was reported to stabilize membranes (Prakash and Prathapsenan, 1988) and to counteract the Na^+^ and Cl^−^ accumulation and induction of the K^+^ efflux (Prakash and Prathapsenan, 1988; Ndayiragije and Lutts, 2006). Polyamine specificity is more and more discussed with dominant polyamines generating a specific signature for the response to a specific stress (Pottosin et al., 2014). Tolerance dependent differences of Put levels under control conditions were independent of subspecies and might be used as potential markers for future breeding efforts.

### Expression levels of genes encoding enzymes involved in polyamine biosynthesis under salinity conditions

To elucidate the molecular basis for changes in polyamine biosynthesis in response to salinity conditions, expression levels of genes encoding enzymes involved in polyamine biosynthesis were analyzed. The expression of many of these genes under different stress conditions and at different growth stages has been analyzed before (Li and Chen, 2000a,b; Kwak and Lee, 2001; Piotrowski et al., 2003; Tian et al., 2004; Hao et al., 2005a; Rodríguez-Kessler et al., 2006). However, the expression profiles of all genes involved in polyamine biosynthesis were previously only studied in rice under drought (Do et al., 2013), but not under salt stress. In *Arabidopsis*, the expression of all genes was investigated under dehydration (Alcázar et al., 2006b), while studies with *Arabidopsis* under salt, dehydration, cold and ABA treatments (Urano et al., 2003) and with maize under salt stress (Rodríguez-Kessler et al., 2006) did not include expressions of *AIH* and *CPA*.

We found expression levels of *ADC2, SAMDC2, SPD/SPM2*, and *SPD/SPM3* induced at 50 mM NaCl in most of the cultivars. Among the three *ADC* genes, mainly *ADC2* was up-regulated. *ADC2* was also found to be stress-induced in *A. thaliana* (Soyka and Heyer, 1999), and mustard (Mo and Pua, 2002). Whereas we found no correlation between the log_2_ fold change of gene expression and the salt sensitivity of the cultivars, another study reported a correlation of the accumulation of an *ADC* transcript with salt stress tolerance in rice (Chattopadhyay et al., 1997). In addition, tolerant cultivars activated two pathways to synthesize Put via arginine and ornithine by higher expression of *ADC2* and *ODC1*, whereas sensitive cultivars only induced one of these genes. Increased synthesis of Put catalyzed by two alternative pathways might be advantageous for the further accumulation of Spm catalyzed by, e.g., *SAMDC2, SPD/SPM2*, and *SPD/SPM3*. Enzyme activity measurements will be necessary to confidently link gene expression data to polyamine pool sizes.

### Comparative analysis of polyamines under drought and salt stress

Our results clearly show that Spm content was significantly increased under both, drought, and salinity conditions, except for tolerant cultivars under drought, which kept their already high initial levels. Under drought stress Spm became the most prominent polyamine, whereas this was only true for the most sensitive cultivars at 100 mM NaCl. The increase in Spm content is consistent with a report by Maiale et al. (2004) for rice under salinity conditions, but contradictory to reports by Krishnamurthy and Bhagwat (1989) for salt and by Liu et al. (2004) for osmotic stress. In these studies, tolerant rice cultivars accumulated higher levels of Spd and Spm, while sensitive rice cultivars showed low levels of these substances and an increase in Put levels. Nevertheless, Spm accumulation seems to be a general feature of plant responses to drought and salinity stress, although its physiological role under stress is still partly unknown. Elevated polyamine levels under salt stress seem to have self-protecting effects due to the modulation of ion channels thereby mediating ion flux homeostasis (Zhao et al., 2007). In roots the immediate effect of polyamines on NaCl-induced K^+^ efflux was dependent on the plant and the polyamine and ranged from beneficial to detrimental (Pottosin et al., 2014). In barley alterations of K^+^-homeostasis, caused by interaction between polyamines and ROS, contributed substantially to genetic variability in salt-sensitivity (Velarde-Buendía et al., 2012).

Together with Spm, Spd may also be involved in the response of plants to stress, e.g., through the induction of stomatal closure (Liu et al., 2000), prevention of chlorophyll loss (Chattopadhyay et al., 2002), stabilization of membranes (Rajasekaran and Blake, 1999) and scavenging of free radicals (Velikova et al., 1998). Several studies reported an accumulation of Spd under salt (Krishnamurthy and Bhagwat, 1989; Basu and Ghosh, 1991) and osmotic stress (Tiburcio et al., 1986; Li and Chen, 2000a). In contrast, decreased Spd levels in response to stress were also reported under salt (Maiale et al., 2004; Sanchez et al., 2005), osmotic (Aziz et al., 1997), and drought stress (Turner and Stewart, 1986). In this study, a reduction of Spd was only observed under drought conditions, whereas under salinity condition the Spd content was unchanged. The ability of Spd to prevent the uptake of Na^+^ and the loss of K^+^ (Chattopadhyay et al., 2002) may suggest that high Spd levels could be more important under salt than under drought stress.

Put levels decreased in our experiments in leaves under drought stress independent of tolerance of the cultivar, while under salinity conditions they were not changed in tolerant and sharply decreased in sensitive cultivars. For salt-sensitive cultivars threefold higher Put levels were observed under control conditions in hydroponic culture compared to cultivation in sand, with Put levels at 50 mM NaCl reaching values comparable to values under control conditions in sand grown plants. Nevertheless this will not affect the comparison between different stress conditions due to the restriction to relative changes in Put levels in comparison to control levels. The decrease of Put levels in all cultivars under drought and in sensitive cultivars under salinity conditions could be caused by the higher substrate need for the Spm synthesis. A strong metabolic canalization of Put into Spm synthesis induced by drought was also described for *Arabidopsis* and *Craterostigma plantagineum* but did not lead to Spm accumulation in *Arabidopsis* due to a Spm-to-Put back-conversion (Alcázar et al., 2011).

A Spm-to-Put back-conversion by polyamine oxidase (PAO) might have also occurred, indicated by the fact that Spm accumulation after salt stress was lower in salt sensitive than in salt tolerant cultivars. On the other hand, Put levels in poplar and tomato did not affect Spm levels, while Spd and Spm levels are inter-dependent (Mattoo et al., 2010). Reduced Put levels could be also reached by the action of DAO yielding pyrroline, H_2_O_2_ and ammonia (Moschou et al., 2012) but avoiding high Put levels, which might be toxic for plants (Slocum et al., 1984; Panicot et al., 2002). For an estimation of degradation processes an analysis of gene expression and enzyme activities of DAO and PAO would be necessary. Due to the involvement of polyamines in stress response as well as programmed cell death the balance between intracellular polyamine concentrations and polyamine catabolism resulting in ROS generation in the apoplast will be crucial for the survival of plants (Pottosin et al., 2014).

Another interesting finding of our study was a descending gradient of Put levels from sensitive to tolerant cultivars already under control conditions. This was also shown previously for the Put levels and drought tolerance of a set of 21 rice cultivars (Do et al., 2013). The higher Put content in sensitive cultivars under control conditions might be a useful tool for breeders to select against sensitivity in breeding programs. In conclusion, we have shown that polyamines are strongly involved in the response of rice to drought and salinity stress. From our results and previous reports we hypothesize that Spm contributes to the drought and salinity tolerance of rice, while the involvement of Spd and Put particularly in salt tolerance still needs to be clarified.

### Comparison of expression of genes encoding enzymes involved in polyamine biosynthesis between salt and drought stress

The comparison of tolerant and sensitive cultivars under the different stress conditions indicated a general up-regulation of *ADC2*, except for sensitive cultivars at 100 mM NaCl. This higher expression could provide an advantage for stress adaptation, e.g., through the *de novo* synthesis of Put as a substrate for longer chain polyamines. Arabidopsis plants over-expressing the *ADC* gene from oat under the control of a stress-inducible promoter were more resistant to dehydration stress associated with an increase of putrescine levels (Alet et al., 2011a). *ADC2* was additionally identified as salt- and drought-induced in *Arabidopsis* in several studies, as reviewed in Alcázar et al. (2006a). Under salt stress at 100 mM NaCl cultivars of the sensitive group seem to compensate the lower *ADC2* induction by the induction of *ODC1*, while tolerant cultivars activated both pathways under salinity which is in contradiction to reports showing that the ADC pathway for polyamine biosynthesis is predominant in higher plants (Birecka et al., 1985; Rajam, 1993; Rodríguez-Kessler et al., 2006). The existence of two alternative routes for the synthesis of Put could be explained by the differential compartmentation of the two enzymes resulting in the specific regulation of different plant processes. With the localization of ADC in chloroplasts, polyamines synthesized via the ADC pathway seem to play a role in maintaining photosynthetic activity (Borrell et al., 1995). Additionally, ADC is thought to be the enzyme primarily responsible for abiotic stress-induced Put accumulation (Galston and Sawhney, 1990; Tiburcio et al., 1997). ODC is found in the nucleus (Slocum, 1991) and mitochondria (Acosta et al., 2005). It has been suggested that ODC is involved in the regulation of cell division/proliferation in growing plant tissues, while ADC is involved in cell expansion (Cohen, 1998).

The transcript level of *AIH* was reported to be up-regulated under drought stress in tolerant as well as sensitive cultivars (Do et al., 2013), but was unchanged in response to salt stress. One report is available for *A. thaliana* under dehydration stress (Alcázar et al., 2006b), where the expression level of *AIH* was slightly increased, in agreement with the results from rice under drought stress.

The *CPA* genes of rice show different expression levels under control conditions both in plants grown in sand and in hydroponic culture. The expression levels of the four genes are related as follows: *CPA*1 > *CPA*2 ≥ *CPA*4 > *CPA*3. Except for a reduction in the expression level of *CPA3* under drought stress (Do et al., 2013), no change was detectable for any *CPA* gene under drought or salt stress, showing that these genes are constitutively expressed. This result is in accordance with the observation that the transcript level of the *CPA* gene in *A. thaliana*, which is similar to *CPA1* from rice, was not altered by osmotic stress (Piotrowski et al., 2003). A drought-dependent repression of *CPA3* in rice has not been reported before.

Several enzymes are involved in the pathway from Put to Spd and Spm, including *SAMDC* and *SPD/SPM*. In response to drought and salt stress, the expression levels of *SAMDC2, SPD/SPM2*, and *SPD/SPM3* were up-regulated with the exception of *SAMDC2* in tolerant cultivars under salt stress. Increased expression of *SAMDC* was also reported in rice (Li and Chen, 2000b; Kawasaki et al., 2001; Rabbani et al., 2003; Shiozaki et al., 2005), wheat (Li and Chen, 2000a), soybean (Tian et al., 2004), and *Arabidopsis* (Alcázar et al., 2006b) under drought and salt, and in maize (Rodríguez-Kessler et al., 2006) for *SPD/SPM* under salt conditions. In addition, a tolerant rice cultivar responded more quickly to salt and reached the highest expression level of the *SAMDC* gene under short-term stress, while the sensitive cultivar reached higher levels after a prolonged time of stress (Li and Chen, 2000b). In our study, a differential regulation of *SAMDC* genes among the different tolerance groups was not observed. However, *SAMDC4* was specifically down-regulated at 100 mM NaCl. Under all conditions and over all tolerance groups an induction of *SPD*/SPM*2, SPD/SPM3*, and *SPD/SPM4* genes was observed. A spermine synthase was also shown to be salt- and drought-induced in *Arabidopsis*, as reviewed in Alcázar et al. (2006a), and a spermine synthase mutant of *Arabidopsis* was shown to be more sensitive to drought and salt stress (Yamaguchi et al., 2007).

**Figure 9:**
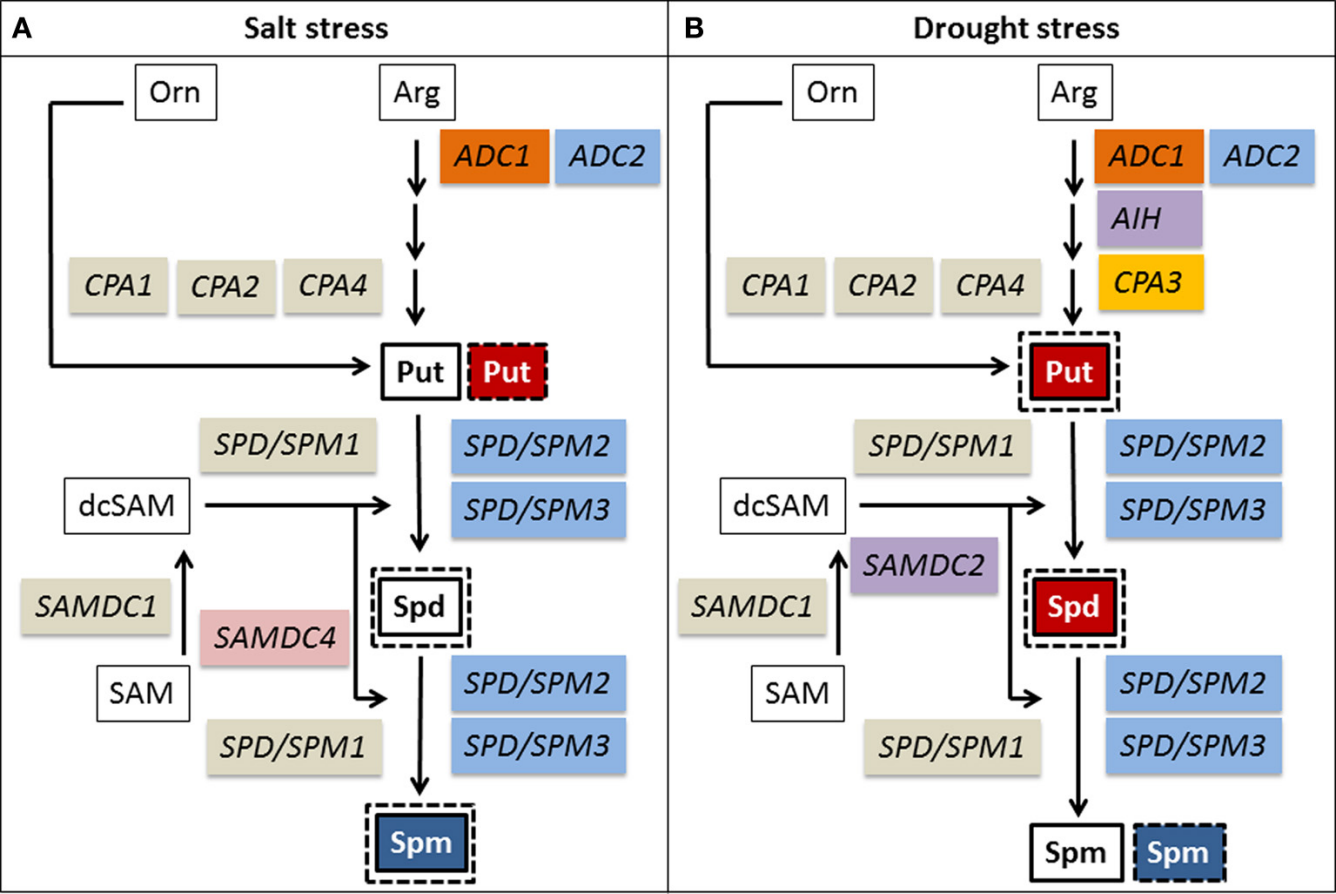
**Changes of polyamine metabolism in response to salt (A) and drought stress (B) in rice leaves of tolerant and sensitive cultivars**. Changes of polyamines are shown for tolerant (solid line) or sensitive cultivars (dotted line). A decrease is shown in red, an increase in blue. Changes in gene expression under stress conditions are color coded as follows: constitutively expressed—gray, induced by drought and salt stress—blue, induced by drought—purple, repressed by salt and drought stress—orange, repressed by drought stress—yellow, repressed by salt stress—pink. Arg, arginine; Orn, ornithine; SAM, S-adenosylmethionine; dcSAM, decarboxylated S-adenosylmethionine.

Changes in gene expression and in polyamine levels in differentially tolerant cultivars under salt or drought stress conditions are summarized in Figure 9. By comparing the gene expression analyses under salt and drought stress in tolerant and sensitive cultivars, three genes that had previously been classified as drought-induced (Do et al., 2013) are now identified as generally stress-induced (*ADC2, SPD/SPM2, SPD/SPM3*), whereas *AIH* was confirmed as specifically drought-induced. *SAMDC2*, on the other hand, was induced by drought and was only induced by salt stress in sensitive cultivars. *ADC1*, which was previously classified as drought-repressed could now be identified as generally stress-repressed, whereas *CPA3* was confirmed as specifically drought-repressed. Only *SAMDC4* could be identified as salt-stress repressed gene. Five genes that were previously classified in drought stress experiments (Do et al., 2013) as constitutively expressed (*CPA1, CPA2, CPA4, SAMDC1, SPD/SPM1*) were confirmed under salt stress.

### Conflict of interest statement

The authors declare that the research was conducted in the absence of any commercial or financial relationships that could be construed as a potential conflict of interest.

## Abbreviations

ABA: abscisic acid
ADC: arginine decarboxylase
AIH: agmatine iminohydrolase
CPA: N-carbamoylputrescine amidohydrolase
ODC: ornithine decarboxylase
Put: putrescine
qRT-PCR: quantitative RT-PCR
ROS: reactive oxygen species
SAMDC: S-adenosyl-methionine decarboxylase
SPD: spermidine synthase
Spd: spermidine
SPM: spermine synthase
Spm: spermine.
